# The improvement of the lower limb exoskeletons on the gait of patients with spinal cord injury: A protocol for systematic review and meta-analysis

**DOI:** 10.1097/MD.0000000000028709

**Published:** 2022-01-28

**Authors:** Xiali Xue, Xinwei Yang, Huan Tu, Wanna Liu, Dezhi Kong, Zhonghe Fan, Zhongyi Deng, Ning Li

**Affiliations:** a Institute of Sports Medicine and Health, Chengdu Sport University, Chengdu, Sichuan Province, China; b School of Sports Medicine and Health, Chengdu Sport University, Chengdu, Sichuan Province, China.

**Keywords:** exoskeletons, gait, lower limb, meta-analysis, protocol, spinal cord injury

## Abstract

**Background:**

Spinal Cord Injury is a severely disabling disease. In the process of Spinal Cord Injury rehabilitation treatment, improving patients’ walking ability, improving their self-care ability, and enhancing patients’ self-esteem is an important aspect of their return to society, which can also reduce the cost of patients, so the rehabilitation of lower limbs is very important. The lower limb exoskeleton robot is a bionic robot designed according to the principles of robotics, mechanism, bionics, control theory, communication technology, and information processing technology, which can be worn on the lower limb of the human body and complete specific tasks under the user's control. The purpose of this study was to evaluate the effect of the lower limb exoskeleton on the improvement of gait function in patients with spinal cord injury.

**Methods:**

The following electronic databases will be searched from inception to January 2022: PubMed, the Cochrane Library, Embase, Scopus, EBSCO, Web of Science, China National Knowledge Infrastructure, WanFang Data, Weipu Electronics. In addition, reference lists of the included studies were manually searched to identify additional relevant studies. Randomized controlled trials were collected to examine the effect of lower limb exoskeletons on lower limb functional recovery in spinal cord injury patients. We will consider inclusion, select high-quality articles for data extraction and analysis, and summarize the intervention effect of lower limb exoskeletons on the upper limb function of spinal cord injury patients. Two reviewers will screen titles, abstracts, and full texts independently according to inclusion criteria; Data extraction and risk of bias assessment were performed in the included studies. We will use a hierarchy of recommended assessment, development, and assessment methods to assess the overall certainty of the evidence and report findings accordingly. Endnote X8 will be applied in selecting the study, Review Manager 5.3 will be applied in analyzing and synthesizing.

**Results:**

The results will provide evidence for judging whether lower limb exoskeletons are effective and safe in improving lower limb function in patients with spinal cord injury.

**Conclusion:**

Our study will provide reliable evidence for the effect of lower limb exoskeletons on the improvement of lower limb function in spinal cord injury patients.

**INPLASY registration number:**

INPLASY202180095.

## 1 Introduction

Spinal Cord Injury (SCI) is a severely disabling disease, often resulting in paraplegia or quadriplegia, which affects the sensory, motor, and autonomic functions of patients.^[1,2]^ According to national statistics, the incidence of the disease is increasing year by year. The incidence of non-traumatic spinal cord injury in developed countries is higher than that of traumatic spinal cord injury, with a rate of 9.3 per million inhabitants per year. In the process of SCI rehabilitation treatment, improving patients’ walking ability, self-care ability and self-esteem is an important aspect of their return to society,^[3]^ which can also reduce the cost of patients, among other rehabilitation targets, regaining independent ambulation has been demonstrated to be a high priority for recovery among patients with SCI.^[4]^ So the rehabilitation of lower limbs is very important.^[5]^ The main functions of the lower limbs are standing and walking. For SCI patients, walking ability is directly related to the injury level and ASIA injury grade. However, data from a recent European multicentre study showed that only 4 out of 10 SCI patients were able to walk independently again.^[6]^ It can be inferred that neuromuscular recovery after SCI is very difficult, so the rehabilitation of patients with SCI should be paid more attention to and early intervention of rehabilitation training.

The lower limb exoskeleton robot is a bionic robot designed according to the principles of robotics, mechanism, bionics, control theory, communication technology, and information processing technology, which can be worn on the lower limb of the human body and complete specific tasks under the user's control.^[7,8]^ It is a new method for walking training for patients with lower limb motor dysfunction. Under the supervision of a therapist, walking training assisted by a traditional therapist is replaced by a mobile exoskeleton that can walk on flat ground. Common manufacturers include Re Walk, Mina, Indego, and Ekso.^[9–11]^ The lower limb exoskeleton rehabilitation robot aims at patients with lower limb motor dysfunction. It is a gait rehabilitation exoskeleton that adopts a joint drive for rehabilitation treatment. The advantage of the exoskeleton robot is that the exoskeleton robot can carry out reasonable walking indoors and outdoors, most of the subjects’ sitting balance is improved, and a small part of the subjects’ muscle strength is improved. While there are still some shortcomings, powered exoskeletons could help patients who have been confined to wheelchairs regain the possibility of walking again. Future improvements to exoskeleton robots could make it possible for patients to use them for daily use and exercise.^[12]^

At present, there are more and more studies on the application of lower limb exoskeleton robots in the rehabilitation and training of SCI. Some studies have reported that lower limb exoskeleton robot is helpful for the improvement of lower limb function,^[13,14]^ but some studies believe that lower limb exoskeleton robot is ineffective for the recovery of lower limb function of patients with SCI.^[15]^ Therefore, the purpose of this systematic review and meta-analysis was to determine the efficacy of lower limb exoskeletons in improving gait function in patients with SCI, compared with placebo or other treatments.

## 2 Methods

### 2.1 Study registration

The protocol of our study has been registered with the international platform of the registered systematic review and meta-analysis protocols (INPLASY) database (INPLASY202180095). The protocol is reported strictly according to the Preferred Reporting Items for Meta-Analyses Protocols (PRISMA-P) guidelines.^[16]^

### 2.2 Eligibility criteria

#### 2.2.1 Types of study

We will include randomized controlled trials of the lower limb exoskeleton to improve upper limb function in SCI patients.

#### 2.2.2 Types of participants

All patients with SCI that affects lower limb function, no restrictions will be applied in terms of age, sex, race, country, and disease.

#### 2.2.3 Types of interventions

The experimental group wore a lower limb exoskeleton for rehabilitation training. The control group received a placebo or other treatment techniques.

#### 2.2.4 Types of outcome measures

The improvement indicators of lower limb gait function in patients with SCI mainly included Berg Balance scale; Time up and go test; 10-meter walk test (10MWT); Fugl-Meyer assessment for the lower-extremity (FMA-LE). The main outcome measure is FMA-LE, The secondary outcome measure are Berg Balance scale, Time up and go test, 10MWT.

### 2.3 Search methods for identification of studies

#### 2.3.1 Electronic data sources

The following electronic databases will be searched from inception to January 2022: PubMed, the Cochrane Library, Embase, Scopus, EBSCO, Web of Science, China National Knowledge Infrastructure, WanFang Data, Weipu Electronics. In addition, reference lists of the included studies were manually searched to identify additional relevant studies.

#### 2.3.2 Other resources

Relevant references will be reviewed and screened. In addition, we will use search engines to search related literature on the Internet, these include Google Scholar and Baidu Academic. Moreover, the authors will search ClinicalTrials.gov (https://clinicaltrials.gov/) to obtain related clinical studies. Besides, if necessary, we will contact investigators for relevant results. Additionally, we will manually perform citation searches to avoid missing critical information.

### 2.4 Search strategy

The search is performed by combining subject terms with free terms. The search terms on PubMed are Exoskeleton (e.g., Exoskeletons or Device, Exoskeleton or Devices, Exoskeleton); Spinal Cord Injury (e.g., Cord Trauma, Spinal or Spinal Cord Traumas or Myelopathy, Traumatic); Lower Limb (e.g., Limb, Lower or Extremities, Lower or Membrum inferius); Randomized controlled trials (e.g., randomized or randomized or clinical trials). Combinations of Medical Subject Headings (MeSH) and text words will be used. The same search terms are used in other electronic databases. These search terms are shown in Table 1. Different databases have different characteristics and different retrieval strategies.

**Table 1:** Search strategy for the PubMed database.

Number	Search items
#1	Exoskeleton
#2	Exoskeletons
#3	Device, Exoskeleton
#4	Devices, Exoskeleton
#5	Exoskeleton Devices
#6	Robotic Exoskeleton
#7	Robotic Exoskeletons
#8	Exoskeleton, Robotic
#9	Exoskeletons, Robotic
#10	#1 or #2–#9
#11	Spinal Cord Injury
#12	Cord Trauma, Spinal
#13	Cord Traumas, Spinal
#14	Spinal Cord Traumas
#15	Trauma, Spinal Cord
#16	Traumas, Spinal Cord
#17	Myelopathy, Traumatic
#18	Myelopathies, Traumatic
#19	Traumatic Myelopathies
#20	Traumatic Myelopathy
#21	Injuries, Spinal Cord
#22	Cord Injuries, Spinal
#23	Cord Injury, Spinal
#24	Injury, Spinal Cord
#25	Spinal Cord Trauma
#26	Spinal Cord Transection
#27	Cord Transection, Spinal
#28	Cord Transections, Spinal
#29	Spinal Cord Transections
#30	Transection, Spinal Cord
#31	Transections, Spinal Cord
#32	Spinal Cord Laceration
#33	Cord Laceration, Spinal
#34	Cord Lacerations, Spinal
#35	Laceration, Spinal Cord
#36	Lacerations, Spinal Cord
#37	Spinal Cord Lacerations
#38	Post-Traumatic Myelopathy
#39	Myelopathies, Post-Traumatic
#40	Myelopathy, Post-Traumatic
#41	Post Traumatic Myelopathy
#42	Post-Traumatic Myelopathies
#43	Spinal Cord Contusion
#44	Contusion, Spinal Cord
#45	Contusions, Spinal Cord
#46	Cord Contusion, Spinal
#47	Cord Contusions, Spinal
#48	Spinal Cord Contusions
#49	#11 or #12–#48
#50	Lower Limb
#51	Limb, Lower
#52	Extremities, Lower
#53	Lower Extremities
#54	Limbs, Lower
#55	Lower Limbs
#56	Membrum inferius
#57	Extremity, Lower
#58	#50 or #51–#57
#59	Randomized controlled trial
#60	Randomized
#61	Clinical trial
#62	#59 or #60–#61
#63	#10 and #49 and #58 and #62

### 2.5 Data collection and analysis

#### 2.5.1 Selection of studies

**Figure 1:**
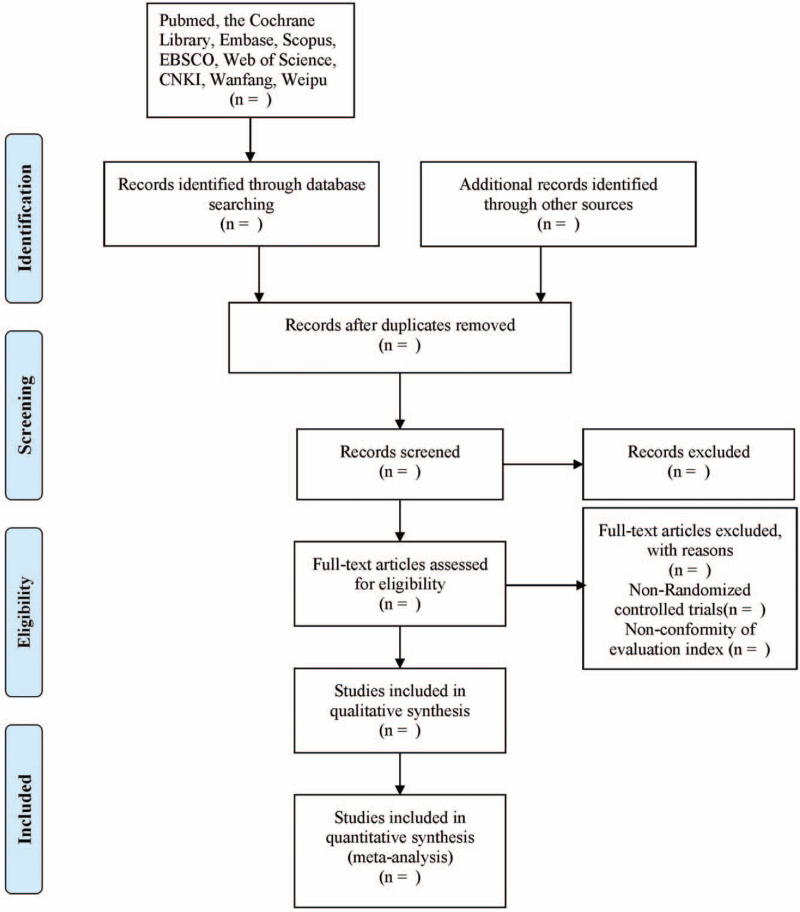
Flow diagram of the study selection process.

Records from databases and other resources will be uploaded to a database created by EndNote X8 software. The abstracts of all studies will be independently screened by the review authors (XLX and XWY). The full text of articles potentially suitable for the review will be obtained for further assessing eligibility based on the inclusion criteria or/and exclusion criteria. The studies that do not fulfill the inclusion criteria will be excluded and listed with reasons for their exclusion. Any disagreement will be resolved by consensus or discussion with a third researcher (NL). The detailed screening process will be shown in the following Preferred Reporting Items for Systematic Reviews and Meta-Analyses Protocols flow diagram (Fig. 1).

#### 2.5.2 Data extraction and management

The other 2 researchers (HT and ZYD) will extract data independently to fill out the predesigned form. The information includes author, country, publication year, methodological quality, characteristics of participants, the details of intervention and comparisons, outcomes, the specific data, results, conclusions, follow-up, adverse events, conflicts of interest, sources of funds, and ethical approval. The extracted data will be cross-checked by the 2 researchers. A third researcher (NL) will be involved in a disagreement occurs. The authors of the studies included will be contacted for further information when necessary.

#### 2.5.3 Assessment of risk of bias in included studies

Two authors independently evaluated the risk of bias of the included studies and cross-checked the results. Disagreements were resolved by consulting a third party. The quality of the included studies was assessed using the Cochrane Collaboration risk assessment tool for randomized controlled trials.^[17]^ The risk of bias (low, unclear, or high) was assessed based on random sequence generation, allocation concealment, blinding of participants and personnel, blinding of outcome assessment, incomplete outcome data, selective reporting, and other biases.

#### 2.5.4 Grading the quality of evidence

We will apply the Grading of Recommendations Assessment, Development and Evaluation (GRADE) method to evaluate the level of confidence in regards to outcomes.^[18,19]^ Two independent reviewers will conduct the assessment. In most cases, disagreements were resolved by discussion between the 2 researchers. If disagreement remained after discussion, a third researcher will be consulted before taking the final decision on the disagreements.

#### 2.5.5 Statistical analysis

The meta-analysis will be performed when an adequate number of sufficiently homogeneous studies are found after data extraction, using Review Manager software. Aggregate-level data will be used for meta-analysis. Data will be pooled using the random-effects model. Continuous outcomes will be presented as mean difference/standard mean difference and 95% confidence intervals.^[20]^ The Review Manager 5.3 software will be used for statistical analysis. The Characteristics of the published studies included will be filled in Table 2.

**Table 2:** The characteristics of the published studies included in the meta-analysis.

Study	Country	Mean age T/C	Sample size T/C	Type of intervention T/C	Duration of trial period	Outcomes	Evaluation index	Follow-up time
								
								
								
								
								
								
								
								
								
								
								

#### 2.5.6 Assessment of heterogeneity

A heterogeneity test will be employed to assess the heterogeneity, which is expressed by the *I*^2^ value. If *I*^2^ < 25%, we will consider it to be small heterogeneity. If 25%<*I*^2^<50%, we will consider it to be moderate heterogeneity. If *I*^2^ ≥ 50%, we will consider it to be large heterogeneity.

#### 2.5.7 Management of missing data

The related corresponding author will be contacted if there are insufficient or missing data. If accurate data is still unavailable after contacting the corresponding author, these studies will be excluded.

#### 2.5.8 Assessment of reporting bias

The authors will evaluate the reporting biases, including publication bias and use funnel plots provided the number of eligible studies exceeds 10. In the case where a reporting bias is signified by the funnel plot's asymmetry, the authors will attempt to explain it.

#### 2.5.9 Subgroup analysis

The subgroup analysis will be conducted if there is substantial heterogeneity between the study results, following items will be considered: exoskeleton type, gender, age, different time points for evaluating outcomes after treatment, and outcome styles.

#### 2.5.10 Sensitivity analysis

We will perform sensitivity analysis based on sample size, research design, heterogeneity quality, methodological quality, and statistical model, excluding trials with low quality, and ensure the stability of analysis results.

#### 2.5.11 Ethical review and informed consent of patients

The content of this study is obtained from the database and does not require ethical approval. We will submit the final research results to a peer-reviewed journal for publication.

## 3 Discussion

Lower limb exoskeleton as a new way of rehabilitation training has broad application space, a large number of clinical evidence, for the incompleteness and incomplete SCI patients, lower limb exoskeleton rehabilitation robot can improve walking ability, effective and safe and reduce pressure sores, pulmonary infection, urinary tract infections, and other complications and improve patients’ dignity, and reduce the cost.^[21,22]^ The improvement of walking ability in SCI patients is certain, but the training should be gradual and persistent.^[23]^ The lower limb exoskeleton may improve cardiopulmonary function, metabolic function, walking ability, functional independence, and quality of life in SCI patients, but the recovery of nerve and lower limb motor function needs further research.

As a new treatment technique for SCI, the lower limb exoskeleton has its irreplaceable advantages and has a great space for further research. The lower-limb exoskeleton robot can provide more accurate assessments and functional exercises that more closely resemble normal movement patterns, and can perform training tasks that require the cooperation of multiple therapists. In addition, some studies have confirmed that the training of exoskeleton robots is safe and feasible, and no serious adverse reactions caused by robot training have been found in clinical trials. However, due to the constraints of subjects, intervention time, and training programs, there is no strong evidence of evidence-based medicine to prove the exact role of robots in the improvement of physical function. Exoskeleton robots can achieve the training effect of conventional rehabilitation means, and their unique advantages can provide better help to patients and therapists, so they can be used as one of the alternatives of training methods. At the same time, the existing research results provide a solid research foundation for the in-depth development of exoskeleton robots, and the technology has a broad space for development. It can be predicted that the lower limb exoskeleton rehabilitation robot will be widely applied in clinical practice in the future.

Lower limb exoskeletons are a kind of rehabilitation auxiliary training device which is easy to wear, high acceptable degree, and painless. Whether the lower limb exoskeleton is suitable for the patient is very important. Studies have shown that the lower limb exoskeleton can improve patients’ lower limb function and realize their dream of walking out of bed again, but its efficacy has not been scientifically and systematically evaluated. The purpose of this study was to evaluate the effect of the lower limb exoskeleton on the recovery of lower limb function in patients with SCI, and hopefully, this review will provide more evidence. This review has some limitations. Different exoskeleton types and different injury types may present a risk of heterogeneity. In addition, the measurements and instruments for the results included in the study may be different.

## Author contributions

**Conceptualization:** Xiali Xue.

**Data curation:** Xinwei Yang, Huan Tu, Zhongyi Deng.

**Formal analysis:** Xiali Xue, Xinwei Yang.

**Funding acquisition:** Ning Li.

**Investigation:** Wanna Liu, Dezhi Kong, Zhongyi Deng.

**Methodology:** Xiali Xue, Xinwei Yang, Zhonghe Fan.

**Software:** Xiali Xue, Huan Tu.

**Writing – original draft:** Xiali Xue.

**Writing – review & editing:** Xiali Xue, Xinwei Yang, Huan Tu, Wanna Liu, Dezhi Kong, Zhongyi Deng, Ning Li.
